# The Joubert Syndrome Gene *arl13b* is Critical for Early Cerebellar Development in Zebrafish

**DOI:** 10.1007/s12264-020-00554-y

**Published:** 2020-08-18

**Authors:** Jian Zhu, Han-Tsing Wang, Yu-Rong Chen, Ling-Ya Yan, Ying-Ying Han, Ling-Yan Liu, Ying Cao, Zhi-Zhi Liu, Hong A. Xu

**Affiliations:** 1 Institute of Life Science, Nanchang University, Nanchang, 330031 China; 2 School of Life Sciences, Nanchang University, Nanchang, 330031 China; 3 Jiangxi Provincial Collaborative Innovation Center for Cardiovascular, Digestive and Neuropsychiatric Diseases, Nanchang, 330031 China; 4 Department of Molecular and Cell Biology, Tongji University School of Life Sciences and Technology, Shanghai, 200092 China; 5 Precise Genome Engineering Center, School of Life Sciences, Guangzhou University, Guangzhou, 510006 China

**Keywords:** Joubert syndrome, *arl13b*, Cerebellum, Development, Granule cell, Purkinje cell, Wnt

## Abstract

Joubert syndrome is characterized by unique malformation of the cerebellar vermis. More than thirty Joubert syndrome genes have been identified, including *ARL13B*. However, its role in cerebellar development remains unexplored. We found that knockdown or knockout of *arl13b* impaired balance and locomotion in zebrafish larvae. Granule cells were selectively reduced in the corpus cerebelli, a structure homologous to the mammalian vermis. Purkinje cell progenitors were also selectively disturbed dorsomedially. The expression of *atoh1* and *ptf1*, proneural genes of granule and Purkinje cells, respectively, were selectively down-regulated along the dorsal midline of the cerebellum. Moreover, *wnt1*, which is transiently expressed early in cerebellar development, was selectively reduced. Intriguingly, activating Wnt signaling partially rescued the granule cell defects in *arl13b* mutants. These findings suggested that Arl13b is necessary for the early development of cerebellar granule and Purkinje cells. The *arl13b*-deficient zebrafish can serve as a model organism for studying Joubert syndrome.

**Electronic supplementary material:**

The online version of this article (10.1007/s12264-020-00554-y) contains supplementary material, which is available to authorized users.

## Introduction

Joubert syndrome (JS) is an autosomal-recessive neurodevelopmental disorder, which is characterized morphologically by the unique molar tooth sign, a complex malformation of the cerebellar vermis and brainstem, with abnormalities of axonal decussation affecting the corticospinal tract and superior cerebellar peduncles [1]. JS is clinically characterized by impaired motor functions and intellectual disability. Most cases of JS are variably associated with impairments of additional organs, including the retina, kidney, skeleton, and liver. More than 30 genes have been identified to cause JS (213300, Online Mendelian Inheritance in Man). Most of the proteins encoded by these genes are located in or near the primary cilium, an organelle found in eukaryotic cells, and this makes JS a typical ciliopathy [1]. Although malformation of the cerebellar vermis is common in JS, the role and mechanisms of the causative genes in cerebellar development have only been reported recently [2, 3].

Mutations of *ARL13B* (HGNC ID 25419) lead to the classical form of JS [4]. The *ARL13B* gene encodes an enzyme belonging to the small GTPase superfamily and this makes it unique among the known causative genes of JS. It has been demonstrated that Arl13b is critical for processes of neural development, such as interneuron migration and placement [5], polarized radial glial scaffold formation [6], and neural tube patterning [7]. Arl13b might also be involved in photoreceptor degeneration and kidney cysts [8, 9]. However, the role of Arl13b in cerebellar development remains a mystery.

The zebrafish has been established as a model organism in studying JS [9, 10]. Here, we explored the role of *arl13b* in early development of the cerebellum and we hope use this powerful model to investigate the pathological mechanisms of JS and help to screen for therapeutic targets.

## Materials and Methods

### Zebrafish Maintenance and Embryo Collection

All zebrafish lines were raised and maintained under a photoperiod of 14 h/10 h (light/dark) at 28.5°C in our facility supplied with filtered fresh circulating water. Wild-type zebrafish of the AB strain and the *arl13b* mutants were kindly provided by Dr. Ying Cao (Tongji University, Shanghai, China) and the *Tg(neurod1:EGFP)* transgenic zebrafish were a gift from Dr. Jing-Wei Xiong (Peking University, Beijing, China). *arl13b* homozygous mutants only survive up to 10 days, so heterozygous mutants were mated to produce homozygous embryos. The homozygous embryos were picked according to the curved tail since this phenotype is almost fully penetrant. The developmental stages of zebrafish were characterized following previously-described morphological criteria [11]. Fish embryos and larvae for *in situ* hybridization and immunostaining were raised in E3 supplemented with 0.003% phenylthiourea from 24 hpf onward to prevent pigment formation. All handling procedures were approved by the Ethics Review Committee at Nanchang University.

### Morpholino Oligonucleotide Microinjection

The morpholino (MO) antisense oligonucleotide blocking the translation of *arl13b* (5’-TTTCCCCCCTAAATGCTTTCACTGG-3’) described previously [9] was purchased from Gene Tools LLC (Philomath, OR, USA). The MO was microinjected into zebrafish embryonic yolk at the one- to two-cell stage.

### Imaging of Zebrafish and Behavior

The morphology of the zebrafish larvae (otolith and body curvature) was imaged at 4 dpf using a Nikon AZ100 microscope (Nikon, Tokyo, Japan). The larvae were raised in Petri dishes and transferred to a new dish at 5 dpf for behavioral analysis. After allowing adaptation to the new environment for 5 min, locomotion was video-recorded for 3 min using a Nikon DS-Fil1 digital camera and processed with NIS-Elements F3.0 (Nikon).

### Whole-Mount *In Situ* Hybridization

We made RNA probes from different templates: PCR products and linearized plasmid DNA. For PCR-based *in situ* templates, we designed PCR primers (listed in Supplemental Table 1) to amplify gene-specific products that contained the T3 promoter sequence, and RNA probes were transcribed *in vitro* using T3-RNA-polymerase. For the linearized plasmid DNA-based *in situ* templates, *shh* (HindIII/T7), *atoh1a* (Nco I /SP6), *ptf1a* (Nco I/SP6), *reelin* (NcoI/SP6), and *roraa* (ApaI/SP6), the last four plasmids were kindly provided by Dr. Sheng-Ping L. Hwang (Institute of Cellular and Organismic Biology, Academia Sinica, Taipei, China). The antisense RNA probes were synthesized with T7 or SP6 RNA polymerase after plasmid DNA linearization. Whole-mount *in situ* hybridization was performed using digoxigenin-labeled antisense RNA probes and alkaline phosphatase-conjugated anti-digoxigenin antibodies (Roche, Mannheim, Germany), as described previously [12]. Embryos were mounted in glycerol, and images were captured using a Nikon AZ100 microscope.

### RNA Isolation and Quantitative Real-Time PCR (qPCR)

Total RNA was extracted from the embryos using RNAiso Plus following the manufacturer’s protocol (Takara, Shiga, Japan). Reverse-transcription reactions were carried out with M-MLV reverse transcriptase (Takara). qPCR assays were performed with SYBR Premix Ex Taq II (Takara) on the Abi-Step-One plus Real-Time PCR system (Applied Biosystems, Foster City, CA, USA). All the primer sequences used for qPCR are listed in Supplemental Table 2. All experiments were conducted at least three times. Student’s *t*-test was applied to analyze the data and *P* < 0.05 was indicated a statistically significant difference.

### Immunostaining

For tubulin antibody staining, we fixed larvae with 2% trichloroacetic acid in PBS for 3 h at room temperature. For other antibodies, we fixed larvae overnight at 4°C in 4% paraformaldehyde (PFA) in PBS supplemented with sucrose (4% w/v) [13]. The larvae were rinsed 3 times in 1×PBST (1×PBS and 0.8% Triton) for 10 min each. Afterward, larvae were dehydrated and rehydrated through graded methanols (50% MeOH once, 100% twice, and 50% once, 10 min each), and then rinsed 3 times in 1×PBST for 5 min each. The larvae were immersed in ice-cold acetone for 20 min at –20 °C, rinsed 3 times in 1×PBST for 5 min each and digested with proteinase K for permeabilization. The concentration and treatment time of proteinase K was determined by the developmental stages of the larvae. After digestion, the larvae were re-fixed with fresh 4% PFA in 1×PBS for 20 min at room temperature (RT), and then rinsed 3 times in 1×PBST for 10 min each. The larvae were blocked with buffer containing 10% serum and 1% dimethylsulfoxide in 1×PBST for 3 h at RT and incubated with primary antibody overnight at 4°C. After rinsing 4 times in 1×PBST for 30 min each, the larvae were incubated with secondary antibody for 4 h at RT. The larvae were rinsed and counterstained with Hoechst 33342 (10 μg/mL) for nuclear staining. The following primary antibodies were used: mouse anti-tubulin (1:1000, T6793, Sigma), mouse anti-parvalbumin (1:1000, MAB1572, Chemicon), goat 3A10 (1:1000, DSHB), and goat anti-GFP (1:500, 600-101-215, Rockland). For fluorescence detection, we used Alexa Fluor 488 donkey anti-goat IgG (H+L) (1:500, A11055, Invitrogen) and Alexa Fluor 488 donkey anti-mouse IgG (H+L) (1:500, A21202, Invitrogen). Fluorescence images of larvae were acquired using an Olympus FV1000 confocal microscope, and Z-series stacks were shown as two-dimensional projections.

### Lithium Treatment

Embryos at 30–37 hpf were incubated in E3 containing LiCl (A100416-0025, Sangon) at a final concentration of 50 mmol/L. Age-matched untreated embryos served as controls.

## Results

### Depletion of *arl13b* Impairs Posture and Locomotion in Zebrafish Larvae

In order to explore the function of ARL13B *in vivo*, we started to investigate whether the neurological features of JS are mimicked in *arl13b* mutant zebrafish, an established model organism [4, 9]. The null mutation of *arl13b* (*arl13b*^*-/-*^), identified in a retroviral insertion screen, led to body axis curvature (Fig. S1) [4, 14]. The curved tail phenotype is almost fully penetrant in homozygous embryos while it is rarely found in wild-type and heterozygous embryos [4]. We took advantage of this readily-recognizable morphological phenotype to pick homozygous mutants for further experiments. The picked embryos were genotyped by PCR which confirmed retroviral insertion into the first exon of the *arl13b* gene. Furthermore, qPCR results demonstrated that the expression of *arl13b* mRNA was almost undetectable in the picked embryos, while it was normally-expressed in wild-type embryos (Fig. S3). The *arl13b*^*-/-*^ mutant larvae [5 days post-fertilization (dpf)] were usually motionless and occasionally moved by trembling and circling. These abnormal movements were not found in wild-type larvae, which swam around freely and elegantly (Movie 1).

**Fig. 1:**
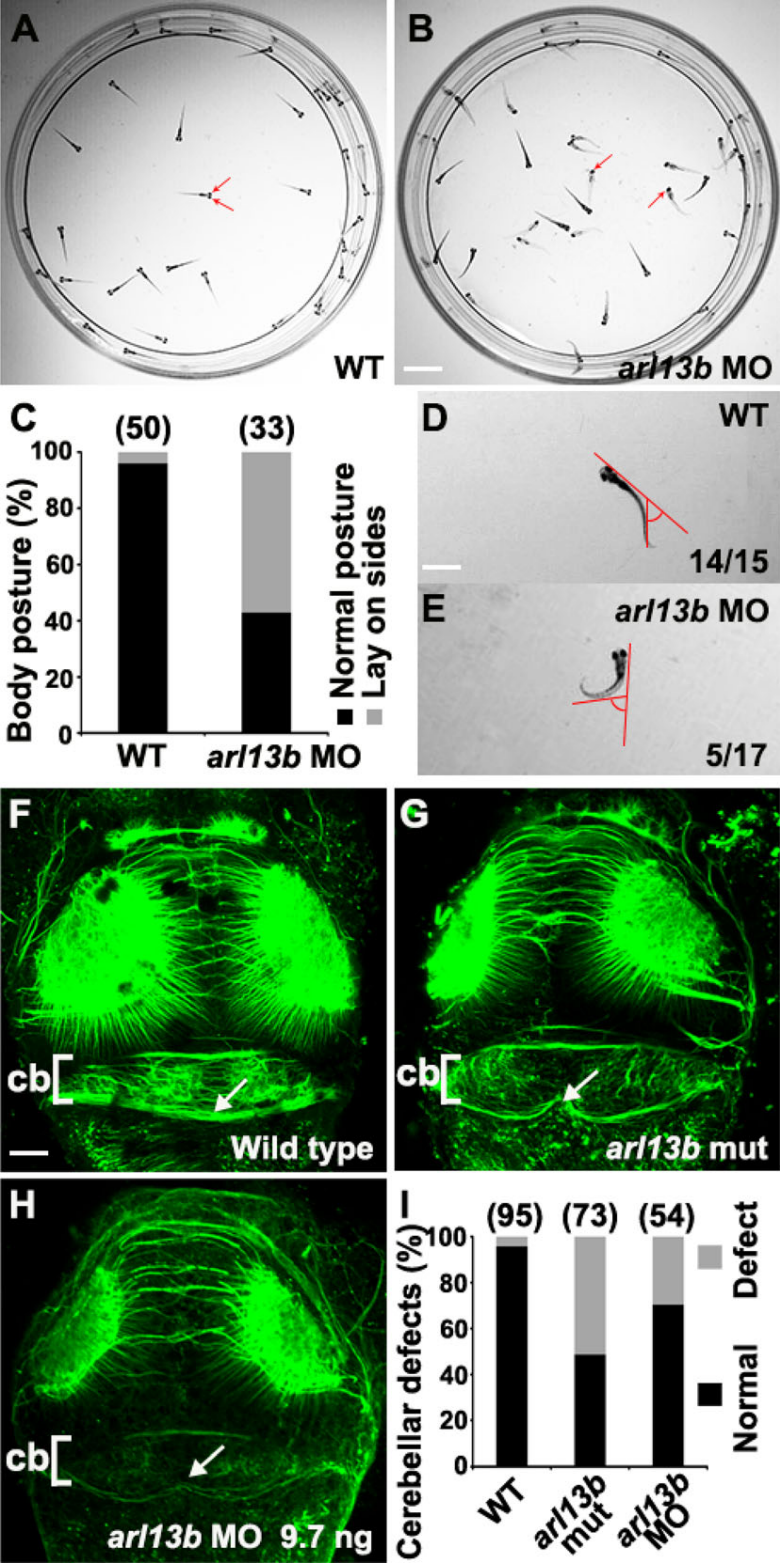
Knockdown of *arl13b* impairs posture, locomotion, and cerebellar morphology in zebrafish larvae. **A** Wild-type sibling larvae remain vertically oriented at 5 days post-fertilization (dpf), with both eyes visible from a top view (arrows). **B** In contrast, larvae injected with *arl13b* MO (subthreshold dose, 7.3 ng) often lie on their side at the bottom of the dish, with only one eye visible (arrows). Note that the body of the subthreshold-dose *arl13b* morphants are relatively straight and only slightly curved. **C** Statistics of the posture of zebrafish larvae at 5 dpf. **D** Wild-type larvae perform stereotyped spontaneous swimming with small bending angles. **E** The *arl13b* morphants (subthreshold dose, 7.3 ng) swim slower and exhibit greater bending angles. **F**–**H** Immunostaining with acetylated tubulin antibody outlines the cerebellum of larvae at 3 dpf. Comparing the dorsal view of wild-type embryos (**F**) with *arl13b* mutants (**G**) reveals morphological defects of the cerebellum (arrows). The cerebellar defects were also present in embryos injected with *arl13b* MO (**H**) (cb, cerebellum). **I** Statistics of the embryos with morphological defects of the cerebellum. The number of embryos examined in each condition is indicated above each column. **A**–**E**, Scale bar 1 cm. **F**–**H**, Scale bar 100 μm.

To assess the phenotype specificity and confirm the locomotor defects, we performed transient knockdown experiments with an antisense morpholino oligonucleotide (MO) designed to specifically block the translation of Arl13b [9]. After injecting 9.7 ng *arl13b* MO into embryos, we observed body curvature and abnormal locomotion like that in *arl13b*^*-/-*^ mutants (Movie 1). Considering that the curvature might interfere with locomotion, we injected a subthreshold dose of MO (7.3 ng) and found that the larvae showed no apparent body axis defects while they still exhibited impairments in posture and locomotion (Movie 1). Wild-type larvae maintained a normal posture (Fig. 1A). In contrast, the subthreshold-dose morphants usually laid on their sides, i.e., lost posture (Fig. 1B, C). As for the swimming patterns, wild-type larvae often exhibited spontaneous swimming characterized by small bending angles (Fig. 1D). However, the subthreshold-dose morphants swam with trembling and exhibited large bending angles (Fig. 1E). The subthreshold-dose results suggested that the posture and locomotion defects in *arl13b* mutants and morphants are due to the depletion of *arl13b* rather than body curvature.

In addition, the sensation of *arl13b* mutants and morphants was compromised; they were not sensitive to a needle poke, while wild-type embryos were sensitive and swam away quickly (Movie 1).

Taken together, the above results suggested that zebrafish deficient in *arl13b* exhibit impairments in posture and swimming pattern, reminiscent of the signs of JS.

### Depletion of *arl13b* Results in Morphological Defects of the Cerebellum

The above behavioral defects suggested the *arl13b*-deficient zebrafish might serve as a good model for studying JS. We first investigated the development of the cerebellum since it is the main cause of the characteristic molar tooth sign in JS. The outline of the cerebellum can easily be distinguished by immunostaining with an anti-tubulin antibody (Fig. 1F). Obvious morphological defects were found in the cerebella of both *arl13b* mutants and morphants, and the antibody-labeled fibers were globally reduced (Fig. 1G, H). The posterior outline of the cerebellum was thinner than that of the wild-type and invaginated anteriorly at the midline, while the anterior outline was relatively normal (Fig. 1F–I). Moreover, the acetyl-tubulin-positive parallel fibers connecting the cerebellar hemispheres were disturbed in *arl13b*-deficient larvae while the commissural axons between the optic tecta remained largely unaffected (Fig. 1F–H). The midline cerebellar defects in *arl13b* mutants and morphants are reminiscent of the midline cerebellar defects in JS patients [2].

Besides the cerebellum, we also checked other neural tissues. *arl13b* is highly expressed in the ventricle and otic vesicle at early developmental stages [9]; the ciliated cells in the inner ear are important for the formation of otoliths [15]. Usually, there are two otoliths in zebrafish (Fig. S1). We found that the otoliths displayed defects in number and size in both *arl13b* mutants and morphants (Fig. S1). The otoliths are critical for proper balance and hearing, and their impairment might contribute to the postural and locomotor defects in *arl13b*-depleted larvae. We also checked motor axons and Mauthner axons, which are involved in locomotion. No morphological defect was observed in either acetylated tubulin-positive motor axons or 3A10-positive Mauthner axons (Fig. S2). We focused on the cerebellum in the subsequent experiments since cerebellar malformation is a major hallmark of JS.

### Depletion of *arl13b* Impairs Granule Cell Progenitors in the Developing Cerebellum

**Fig. 2:**
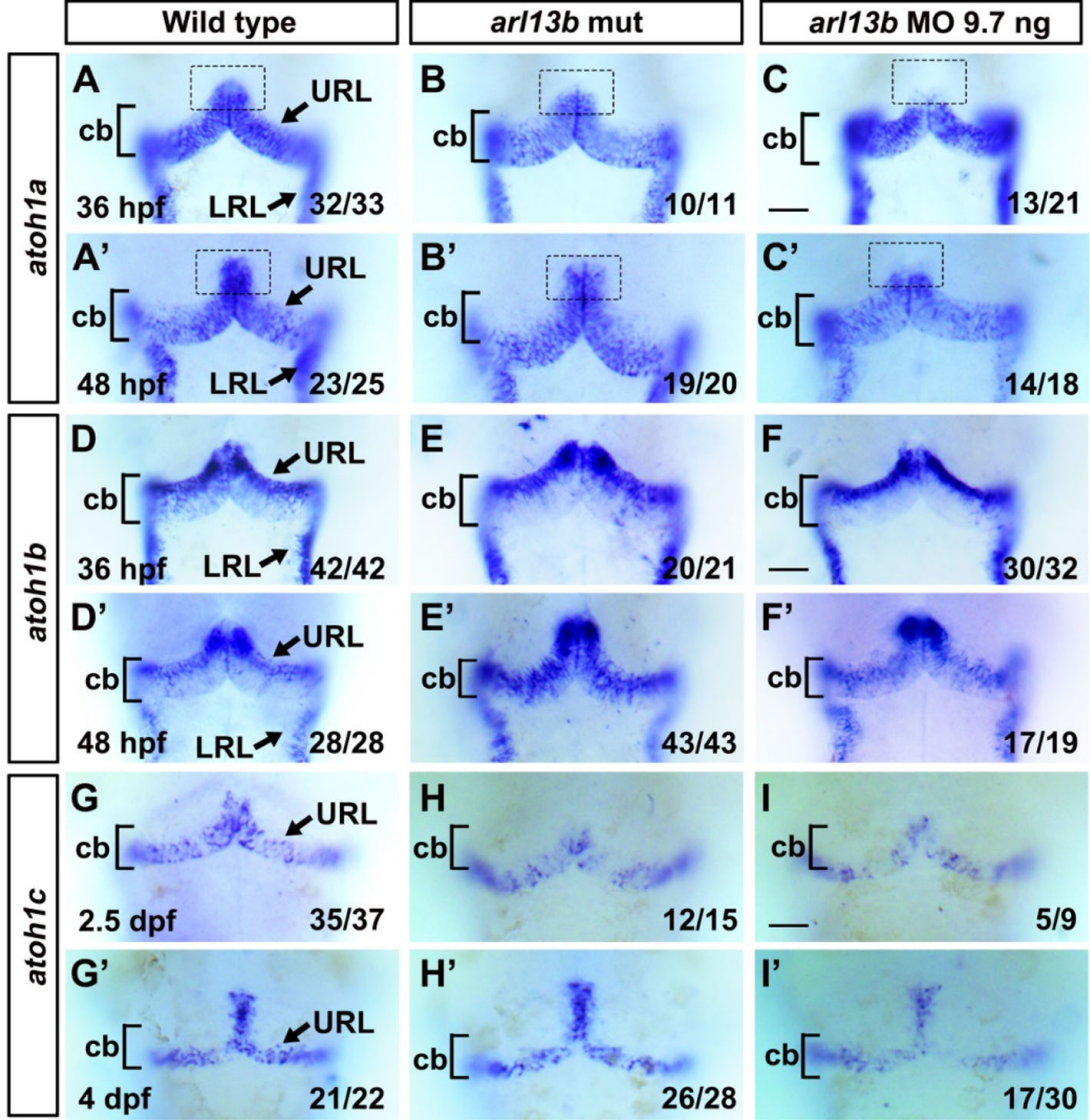
The expression of markers of cerebellar granule cell progenitors is impaired in *arl13b*-deficient embryos. **A**–**I’** Representative images of *in situ* hybridization illustrate that the three paralogues of *atoh1* (*atoh1a*, *1b* and *1c*) are expressed in distinct populations of cerebellar granule cell progenitors. *atoh1a* is expressed in the URL and LRL. Similar expression patterns of *atoh1a* occur in wild-type embryos (**A**) and *arl13b* mutants (**B**) while its expression is absent from the oral dorsomedial URL (dashed box) in *arl13b* morphants (**C**) at 36 hpf and 48 hpf (**A’**–**C’**). The expression patterns of *atoh1b* remained unaffected in *arl13b* mutants and morphants (**D**–**F’**). The expression level of *atoh1c* was decreased in the URL in *arl13b* morphants (**I** and **I’**) (cb, cerebellum; URL, upper rhombic lip; LRL, lower rhombic lip). **A**–**I’**, Scale bar 100 μm.

The morphological defects in *arl13b*-deficient embryos prompted us to further investigate the role of *arl13b* in the development of cerebellar neural circuits. Like mammals, the zebrafish cerebellum is derived from the dorsal part of the anterior hindbrain [16]. The cerebellum is composed of several types of neurons, which are categorized according to their major neurotransmitter, glutamate or GABA. The glutamatergic granule neurons are derived from granule cell progenitors located in the upper rhombic lip (URL). Granule cells are the most abundant type of neuron in the cerebellum. We first examined the expression of the granule progenitor cell marker *atoh1* using whole-mount *in situ* hybridization. In zebrafish, there are three paralogues of *atoh1* – *1a*, *1b*, and *1c* – which are expressed sequentially in overlapping but distinct granule cell progenitors within the rhombic lip [17, 18]. In wild-type embryos, *atoh1a* was strongly expressed in the URL and the lateral rhombic lip (Fig. 2A). In some *arl13b* morphants, *atoh1a* was absent from the anterior dorsomedial URL along the midline (Fig. 2C). The specific absence of *atoh1a* was maintained at 48 hours post-fertilization (hpf) (Fig. 2A’–C’), suggesting that the phenotype could not be due to developmental delay. No detectable change of *atoh1b* was found (Fig. 2D–F’), indicating that the absence of *atoh1a* dorsomedially was specific and not due to defects in cerebellar structure. The expression level of *atoh1c* was decreased in *arl13b* morphants at 2.5 dpf and 4 dpf (Fig. 2G–I’). In *arl13b* mutants, *atoh1c* was decreased slightly at 2.5 dpf (Fig. 2H). The expression defects of *atoh1a* and *atoh1c* were more apparent in *arl13b* morphants than in mutants. This could be due to the maternally-deposited *arl13b* mRNA in null mutants which might mask the early defects caused by Arl13b deficiency [9]. The MO blocks the translation of *arl13b* mRNA, including the maternally-derived mRNA, so the phenotypes in morphants are more penetrant than in mutants [9]. However, potential off-target effects of the MO could not be excluded although it seems unlikely since most phenotypes have been reported in *arl13b* mutants [9] (and our data).

We performed quantitative PCR (qPCR) using whole body tissues to verify the above results. *atoh1c* was consistently down-regulated in *arl13b* mutants from 36 hpf to 4 dpf (Fig. S3). However, *atoh1a* and *1b* were up-regulated at 36 hpf but down-regulated at later stages, when comparing *arl13b* mutants with the wild-type (Fig. S3). It should be noted that we used the whole zebrafish body and the qPCR results represented the mixed expression of all tissues. According to our *in situ* hybridization results and those of others [17, 18], *atoh1c* is mainly expressed in the URL (Fig. 2). The decrease of *atoh1c* in *arl13b* mutants revealed by qPCR indicated that *atoh1c* is reduced mainly in the URL and confirmed our *in situ* hybridization results.

*atoh1c* and *1a* are critical for the full complement of granule cells in the corpus cerebelli (CCe), a structure homologous to the mammalian cerebellar vermis [17, 19]. The decrease of *atoh1a* and particularly *atoh1c* revealed by *in situ* hybridization and qPCR in *arl13b*-deficient embryos might interfere with the development of granule cells in the CCe.

**Fig. 3:**
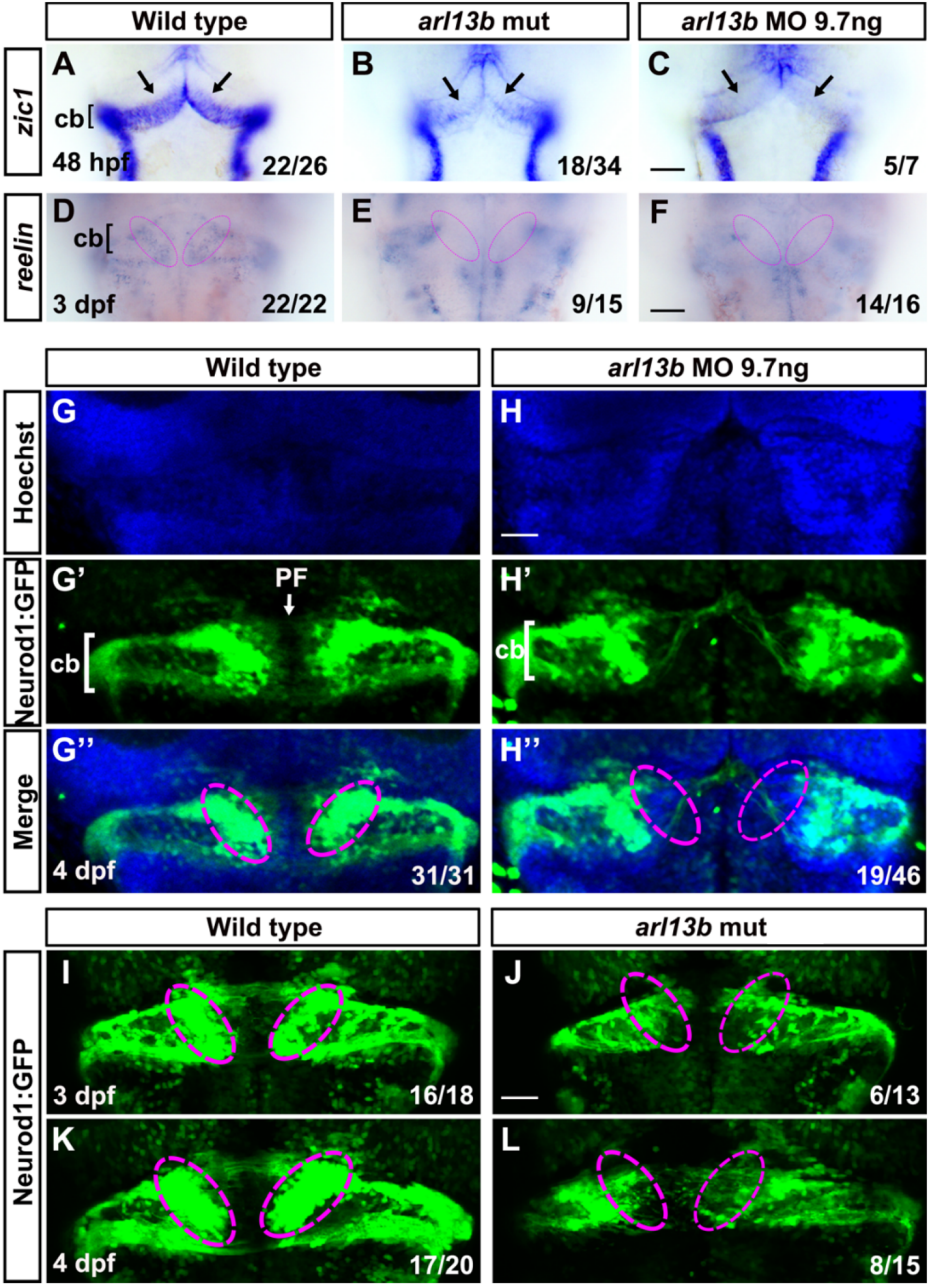
Disruption of *arl13b* impairs the development of cerebellar granule cells. **A** Expression of the granule cell progenitor marker, *zic1*, in the cerebellum of wild-type embryos at 48 hpf revealed by *in situ* hybridization. **B, C** Expression of *zic1* is reduced in the URL of both *arl13b* mutant and morphant embryos compared with wild-type embryos. Note that *zic1* expression is severely reduced in the dorsomedial subregions of the URL (arrows). **D** The expression of the differentiated granule cell marker, *reelin*, in the cerebellum of wild-type embryos at 3 dpf. **E, F** Expression of *reelin* is reduced in the cerebellum of both *arl13b* mutant and morphant embryos. Note that in some embryos *reelin* expression is almost absent in the dorsal medial subregions of the cerebellum (colored ovals). **G**–**G’’** In *Tg(neurod1:eGFP)* transgenic embryos, GFP+ granule cells are grouped into three clusters, the dorsomedial (dashed ovals), dorsoposterior, and ventrolateral subdivisions. **H**–**H’’** The pattern of GFP+ granule cells is dramatically altered in the cerebellum, and particularly in the dorsomedial cerebellar subdivisions (dashed ovals) are severely affected in *arl13b* morphants. The parallel fibers connecting the two hemispheres are disrupted in *arl13b* morphants. Dorsal views of the embryos are shown. **I**–**L** Malformations of the dorsomedial cerebellar subdivisions (dashed ovals) and parallel fibers are also present in *arl13b* mutants both at 3 dpf and 4 dpf. cb, cerebellum; PF, parallel fiber. **A**–**F**, Scale bar 100 μm. **G**–**L**, Scale bar 50 μm.

Zic1 has been shown to be involved in mouse granule cell proliferation [20]. We found that *zic1* was expressed in the URL cells in wild-type zebrafish at 48 hpf while it was dramatically down-regulated in *arl13b* morphants and mutants (Fig. 3A–C).

These results suggested that Arl13b participates in cerebellar development by regulating the development of granule cell progenitors.

### Depletion of *arl13b* Impairs Granule Neurons Specifically in the Corpus Cerebelli

**Fig. 4:**
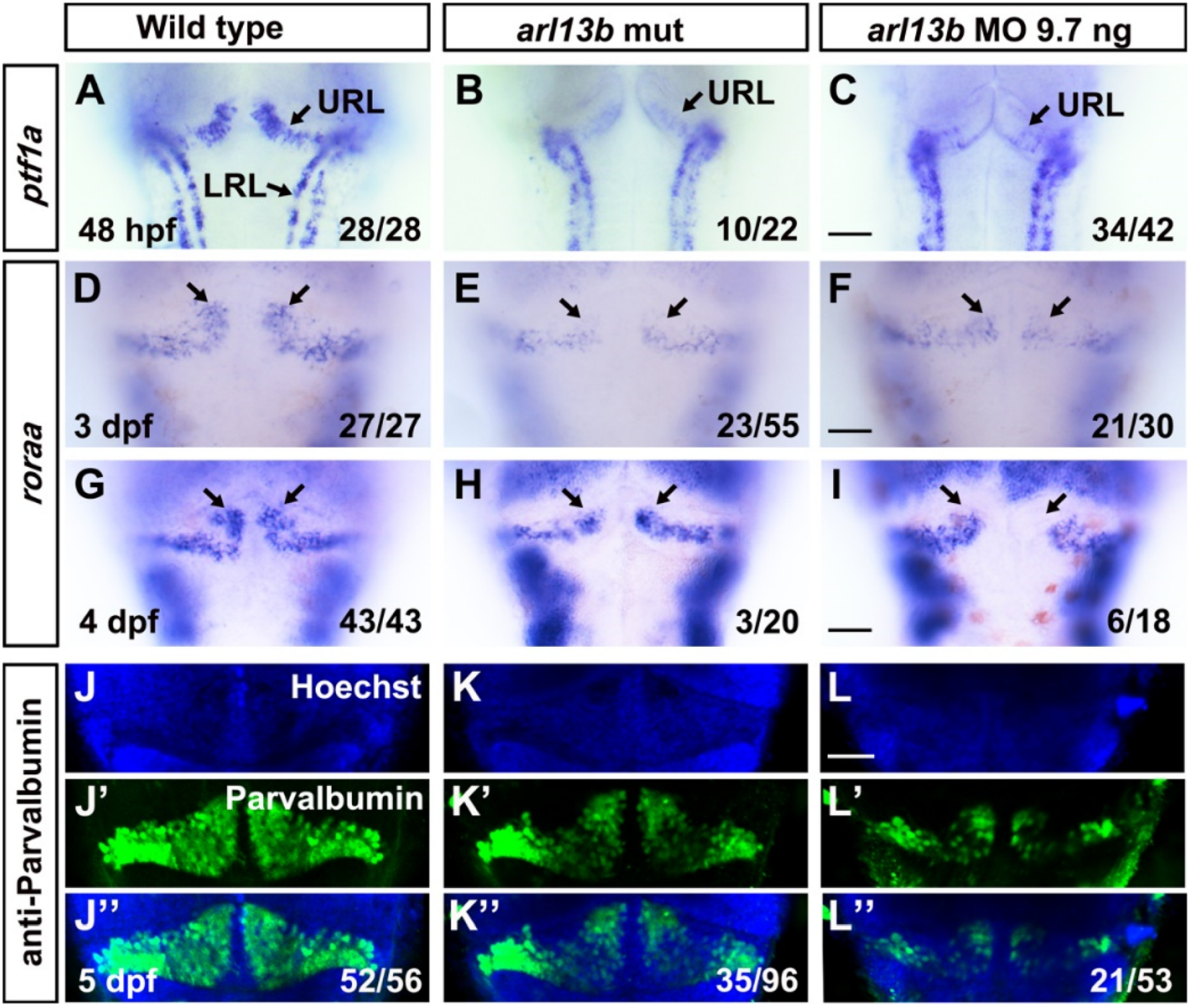
Disruption of *arl13b* reduces both precursor and differentiated cerebellar Purkinje cells. **A**–**I** Representative images of *in situ* hybridization using anti-sense probes against *ptf1a* and *roraa* to label Purkinje precursors and differentiated cells, respectively. The dorsomedial clusters of precursor of Purkinje cells and differentiated Purkinje neurons are selectively reduced (arrows). **J**–**L’’** Immunostaining of mature Purkinje neurons using anti-parvalbumin antibody reveals that the dorsomedial Purkinje neurons are reduced in *arl13b*-deficient embryos. **A**–**I**, Scale bar 100 μm. **J**–**L’’** Scale bar 50 μm.

The impairment of granule cell progenitors might interfere with the subsequent development of granule neurons, so we next examined the differentiation of granule neurons. We found that *reelin*, a marker of differentiated granule cells [21], was markedly decreased in the cerebellum of *arl13b*-deficient embryos, particularly in the dorsomedial subregions (Fig. 3D–F). It has been demonstrated that *NEUROD1* is expressed in immature and mature cerebellar granule neurons in both mammals and zebrafish [18, 22]. We found that *neurod1* was absent from the dorsomedial cerebella of *arl13b* morphants (Fig. S4) while the dorsolateral neurons were not affected, similar to the expression of *reelin*. We further examined the development of cerebellar granule cells using the transgenic line *Tg(neurod1:EGFP)* [23]. In these embryos, GFP was expressed in three main clusters: two close to the midline, the dorsomedial and dorsoposterior granule cells, and one distant from the midline, the ventrolateral granule cells (Fig. 3G–G’’). The patterns of the *Tg(neurod1:EGFP)* GFP+ granule cells resembled the granule cells labeled by *Tg(gata1:GFP)* [19]. Knocking down *arl13b* in *Tg(neurod1:EGFP)* embryos caused a global reduction of GFP+ granule neurons compared to the control transgenic embryos. Particularly, the dorsomedial clusters were severely affected and, in some embryos, these clusters were totally absent (Fig. 3H–H’’). The ventrolateral and dorsoposterior clusters were still present though with reduced numbers of GFP+ neurons (Fig. 3H–H’’). These results were consistent with the expression pattern of *neurod1* detected by *in situ* hybridization (Fig. S4). The dorsomedial subdivision of granule neurons populate the CCe [17, 19]. The defects of CCe granule cells in *arl13b*-deficient zebrafish resembled the defects in the cerebellar vermis found in JS patients. *Tg(neurod1:EGFP)* labeled some parallel fibers at the midline of cerebellum (Fig. 3G’). Upon *arl13b* knockdown, the parallel fibers were dramatically disrupted (Fig. 3H’), similar to those found by anti-tubulin immunostaining (Fig. 1F–I). These phenotypes were also found in *arl13b* mutants (Fig. 3I–L). The *arl13b* mutants were crossed with *Tg(neurod1:EGFP)* fish and transgenic labeled *arl13b* homozygotes were picked. The selective malformation of CCe granule cells and disruption of parallel fibers were frequently observed in *arl13b* mutants both at 3 dpf (Fig. 3I, J) and 4 dpf (Fig. 3K, L).

The qPCR results revealed that the markers of differentiated granule cells *calbindin 2a* (*calretinin*, *calb2a*) and GABA receptor alpha 6a (*gabra6a*) were dramatically reduced in *arl13b* mutants (Fig. S3). These results suggested that not only the proliferation and migration but also the differentiation of granule cells is impaired in *arl13b*-deficient embryos.

### Depletion of *arl13b* Impairs the Development of Cerebellar Purkinje Cells

Both the function and development of the cerebellum are dependent on the cooperation between granule neurons and Purkinje cells, two major neuronal types in the cerebellum, so we further investigated the role of *arl13b* in the development of Purkinje cells. *ptf1a* was used as a marker to label the precursors of Purkinje cells by *in situ* hybridization and it was found to be expressed in the ventricular zone of wild-type embryos at 48 hpf (Fig. 4A). In *arl13b* mutant and morphant embryos, the expression level of *ptf1a* was dramatically and selectively reduced in the dorsomedial ventricular zone, while it was relatively normal in the ventrolateral regions (Fig. 4B, C). The phenotype of morphants was more penetrating than that of mutants, as found in granule cells. These results demonstrated that the progenitors of Purkinje cells are selectively reduced dorsomedially in *arl13b*-deficient embryos. We further examined differentiating Purkinje cells using *roraa* as a marker [24]. This revealed that the dorsomedial clusters of differentiating Purkinje cells were dramatically reduced, while the ventrolateral clusters were only mildly reduced in *arl13b*-deficient embryos at 3 dpf (Fig. 4D–F). This is reminiscent of the selective reduction of dorsomedial granule cell clusters (Fig. 3). A similar decrease of dorsomedial clusters was observed at 4 dpf (Fig. 4G–I). As Purkinje cells differentiate and mature, early distinct clusters of Purkinje cells merge together, become unified, and form a continuous layer spanning the mediolateral width of the cerebellum [25]. Immunostaining with an antibody against parvalbumin was used to label differentiated Purkinje cells and demonstrated that they were dramatically reduced globally in the cerebellum at 5 dpf, including both the dorsomedial and ventrolateral clusters (Fig. 4K–L’’). qPCR results revealed that the markers of immature and mature Purkinje cells *roraa*, *aldoca*, and *pvalb7* were reduced (Fig. S3D), consistent with the immunostaining results.

The global reduction of mature Purkinje cells differed from the specific reduction of dorsomedial clusters of granule cells at the late larval stage. The early specific dorsomedial defects of both Purkinje progenitors and granule progenitors are likely due to unknown common defects in the mechanisms required and partly shared by the two types of neural progenitor.

### Depletion of *arl13b* Reduces *wnt1* Expression in the Developing Cerebellum

Arl13b has been demonstrated to be associated with Hedgehog signaling pathways [7, 26, 27], so we examined the expression of Hedgehog signaling components by *in situ* hybridization and qPCR. *shh* was not detected in the cerebellar anlage at 24 hpf although it was ventrally expressed along the neural tube (Fig. S5). No detectable change of *shh* expression in the cerebellum was observed in *arl13b* morphants, although its expression in the zona limitans intrathalamica was reduced dorsally (Fig. S5). The expression level of the hedgehog receptor *patched1* was relatively low (Fig. S5). It was hard to tell whether it was expressed in the cerebellum in wild-type embryos at 30 hpf as well as whether its expression level or pattern changed. We then used qPCR to assess the expression levels of Shh signaling components. The expression of the receptors *smoothen* (*smo*) and *patched1* (*ptch1*), and the Hedgehog signaling components *gli2a*, *gli2b*, and *gli3* were dynamically regulated in the early stages of cerebellar development (Fig. S5). These results suggested that Hedgehog signaling components are altered globally in *arl13b* mutants while no detectable change occurs early in cerebellar development.

**Fig. 5:**
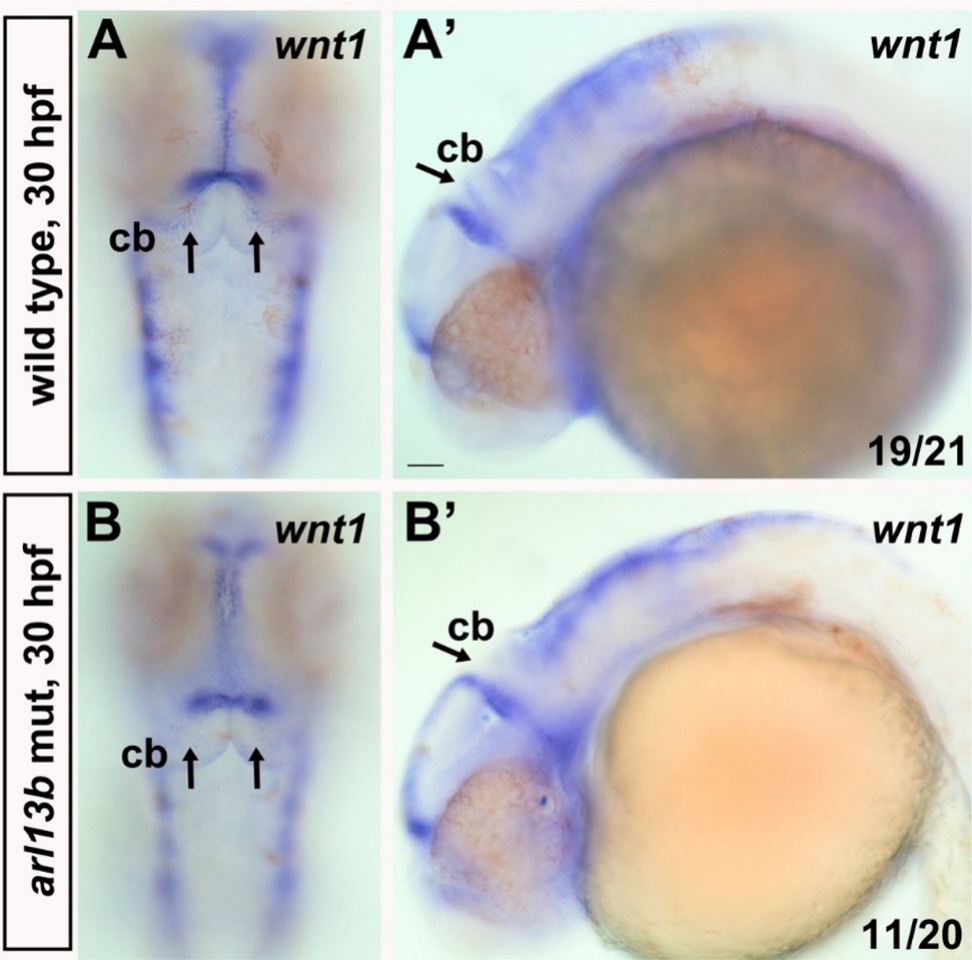
Wnt1 is selectively down-regulated in the cerebellum of *arl13b* mutants. *wnt1* is transiently expressed in the cerebellum of WT embryos (**A, A’**) (arrows) while it is dramatically reduced in *arl13b* mutants (**B, B’**). A and B, dorsal view; A’ and B’, lateral view. Note that the expression of *wnt1* is selectively decreased in the cerebellum while its expression at the midbrain–hindbrain boundary is intact. **A**–**B’**, Scale bar 100 μm.

Wnt signaling is also critical for cerebellar development [28] and cooperates with Hedgehog signaling to regulate cerebellar olig2+ cell development [29]. Intriguingly, recessive mutations of human *WNT1* result in hypoplasia of the cerebellum, particularly of the vermis, suggesting a conserved role of WNT1 in vermis development [30]. Besides being mainly expressed at the midbrain–hindbrain boundary, in mice *wnt1* is also transiently expressed in the URL [31], from which many cerebellar neurons are derived. The transient expression of *wnt1* in the URL is conserved in zebrafish [29]. We found that *wnt1* was transiently expressed in the dorsal cerebellum at 30 hpf in wild-type embryos (Fig. 5A). Its expression was strikingly reduced and almost absent from the cerebellum of some *arl13b* mutants (Fig. 5B), indicating that Wnt signaling is disrupted early in cerebellar development.

### Activating Wnt Signaling Partially Rescues Cerebellar Defects in *arl13b* Mutants

**Fig. 6:**
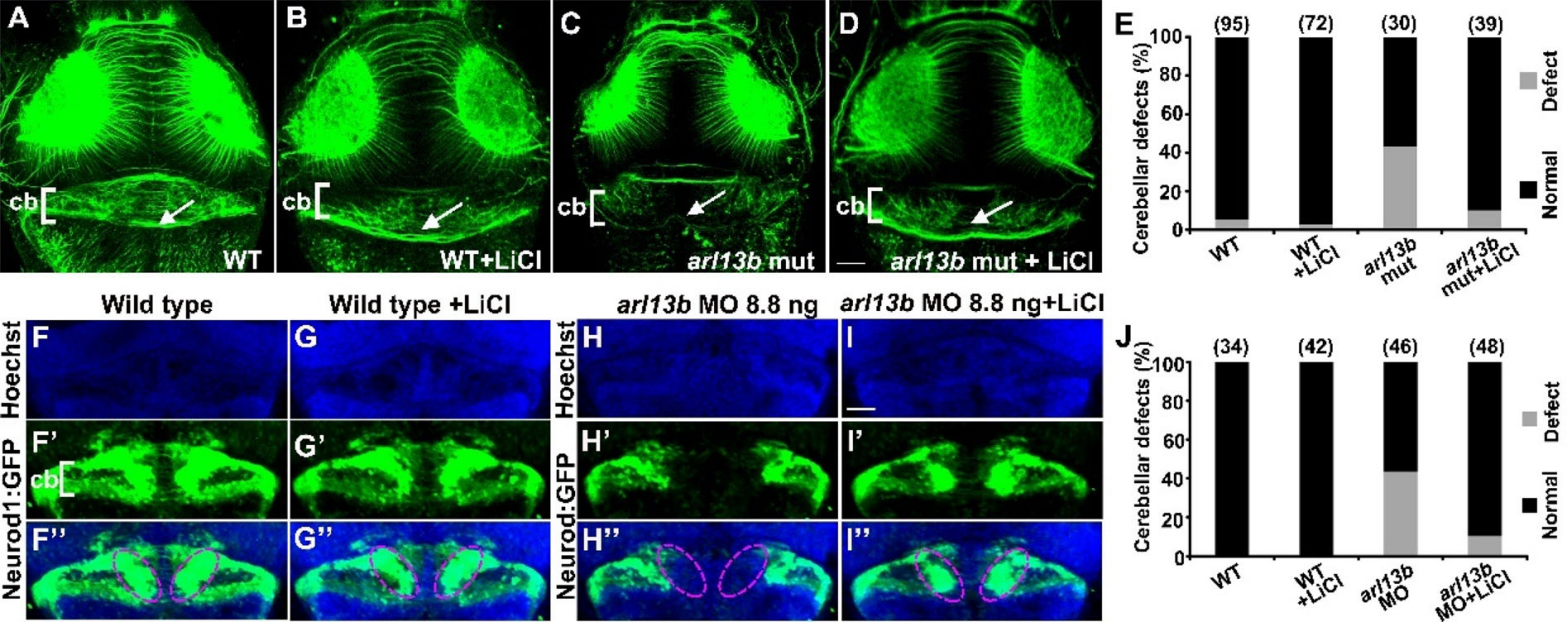
Treating the *arl13b* mutants with lithium mitigates the morphological defects in the cerebellum. **A**–**D** Representative images of embryos treated with 50 mmol/L LiCl at 30–37 hpf, fixed at 3 dpf, and immunostained with anti-tubulin antibody to reveal cerebellar morphology. Treatment of wild-type embryos with Li^+^ does not affect the cerebellar morphology (**A, B**) (arrows). The morphological defects of the cerebellum in *arl13b* mutants treated with Li^+^ are partially rescued (**C, D**) (arrow). **A**–**D,** Scale bar 100 μm. **E** Statistics revealing that the proportion of *arl13b* mutant embryos with cerebellar defects is dramatically decreased by LiCl treatment. **F**–**I’’** Representative images of *Tg(neurod1:EGFP)* transgenic embryos used to label granule cells. Treating wild-type transgenic embryos with Li^+^ causes no defect (**F**–**G’’**) (dashed ovals). Treating *arl13b* morphant transgenic embryos restores the dorsomedial cluster of granule cells (**H**–**I’’**) (dashed ovals). **F**–**I’’**, Scale bar 100 μm. **J** The proportion of *arl13b* morphant embryos with cerebellar defects in the dorsomedial clusters is dramatically reduced by LiCl treatment.

We started to explore whether activating Wnt signaling with lithium, an agonist of Wnt signaling, can mitigate the cerebellar defects in *arl13b*-deficient embryos. The dose of lithium chloride (LiCl) was optimized for different developmental stages. We found that treating the embryos with 50 mmol/L LiCl at 30–37 hpf reduced the cerebellar structural defects in *arl13b* mutants. The wild-type embryos treated with LiCl at the same time showed no detectable cerebellar defects (Fig. 6A–E). We further examined the rescue effects of LiCl with *Tg(neurod1:EGFP)* embryos and found that the dorsomedial reduction of granule cells caused by *arl13b* knockdown was mitigated by LiCl treatment (Fig. 6F–J).

## Discussion

Most published papers study the role of JS genes during late embryonic or postnatal development of the cerebellum, though the molar tooth sign can be detected early in the first trimester [32]. Moreover, these studies mainly focus on cerebellar granule cells. In this study, we found that the disruption of *arl13b* in zebrafish larvae leads to early cerebellar malformations and defects in both granule and Purkinje cell progenitors. The early developmental patterning of cerebellar granule neurons is compromised particularly in the dorsomedial subregions of the CCe, a structure homologous to the mammalian cerebellar vermis. This phenotype is reminiscent of hypoplasia of the cerebellar vermis in JS. Molecular and cellular studies revealed that the early development of the progenitors of both granule cells and Purkinje cells are selectively altered in *arl13b*-deficient embryos. Arl13b may be involved in regulating a network of signaling pathways, including Wnt and Atoh1. Treating the *arl13b* mutants with Li^+^, an agonist of Wnt signaling, partially rescued the cerebellar defects.

Our finding that granule neurons are reduced specifically in the dorsomedial subregions of the CCe in *arl13b*-deficient zebrafish larvae is consistent with the enrichment of *arl13b* expression in the ventricle at early stages, from 25 hpf to 40 hpf and later [9, 33]. This finding is also consistent with reports that granule neurons are reduced along the anteroposterior but not the mediolateral axis in mouse mutants of the cilia genes *Kif3a*, *Ift8*, or *Rpgrip1l* [34, 35]. The reduction of granule neurons in these mutants could be caused by proliferation defects in granule progenitors [34, 35]. Although the cerebellar granule neurons in *Ahi1-* or *Cep290-* knockout mice are only slightly affected, there are significant proliferation defects of granule cells at an early developmental stage (E16.5)[2]. Proliferation defects of granule progenitors seem to be common in these cilia gene mutants [3, 34, 35]. The reduction of granule neurons in *arl13b*-deficient zebrafish was likely caused by proliferation defects in granule progenitors since the expression pattern of *atoh1a* was altered and *atoh1c* was dramatically down-regulated (Fig. 2 and S3). Atoh1 is known to be critical for cerebellar granule neurogenesis in the mouse [36] and for the proliferation of granule cells in zebrafish [18]. *atoh1c*-derived cells contribute to the majority of granule neurons in the CCe, while a minority is derived from *atoh1a* progenitors [17, 18]. The dramatic down-regulation of *atoh1c* and the selective hypoplasia of the CCe in *arl13b*-deficient embryos are consistent with the critical role of *atoh1c* in populating granule neurons in the CCe [17]. The dramatically decreased expression of *zic1* and *neurod1* specifically in the dorsomedial subregions of the cerebellum (Figs. 2 and S4) is also consistent with the proliferation defects of granule progenitors since both genes are implicated in granule cell proliferation [18, 20]. We immunostained the embryos using anti-pH3 antibody, a marker of cell proliferation, and found reduced fluorescence intensity of pH3-positive cells in the cerebellum of *arl13b* mutants (Fig. S6). These results are consistent with potential proliferation defects of granule cells in the cerebellum.

It has been shown that Arl13b regulates the migration and location of interneurons [5]. The specific hypoplasia of the dorsomedial subregions of the CCe in *arl13b*-deficient zebrafish could also be due to migration defects in granule cell precursors. It has been demonstrated that the URL generates different populations of granule cell precursors along its mediolateral axis. These precursors migrate along different routes and form different functional compartments of the mature cerebellum: the eminentia granularis and the CCe [19]. Atoh1 has been shown to be critical for the migration of granule cell precursors out of the URL in mice and particularly Atoh1c in zebrafish [17, 36]. The reduction of *atoh1c* expression in *arl13b*-deficient zebrafish could lead to migration defects of the granule cell precursors. The migration defects might impair the differentiation of granule neurons since several markers of granule neurons are altered, particularly *neurod1*. The absence of *Tg(neurod1:EGFP)*+ cells in the dorsomedial domain of the cerebellum in *arl13b* mutants differs from that seen in *atoh1c* mutants [17], in which *neurod1* is expressed in most of the *Tg(atoh1c:kaede)*+ granule cell progenitors, including the dorsomedial cells along the midline. These results suggest that depletion of *arl13b* also disrupts other signaling pathways besides Atoh1.

Wnt1 is transiently expressed in the cerebellum of zebrafish [29] (Fig. 5) and mouse [31] and its mutations in humans result in hypoplasia of the cerebellar vermis [30], indicating that Wnt signaling is a potential mechanism underlying the pathology of *arl13b* mutant zebrafish and humans. Besides *wnt1*, other *wnt* genes have also been detected in the cerebellum, such as *wnt3*, *wnt7a* and *wnt10b* [37], which might be involved in early cerebellar development [3, 38, 39]. Further study is required to investigate whether they are also regulated by Arl13b. The Wnt downstream signaling components β-catenin and Gsk3β regulate the transcription and protein stability of Atoh1 [40, 41]. Wnt1 has been proposed to regulate Atoh1 expression in the developing cerebellum [31]. These reports are consistent with our findings that *atoh1c* and *1a* expression is reduced in *arl13b*-deficient embryos (Figs. 2 and S3). Moreover, wnt1 is also expressed at the midbrain–hindbrain boundary and it has been speculated to contribute to the development of the cerebellum. Whether the boundary Wnt1 also contributes to the cerebellar defects in *arl13b*-deficient embryos needs further experiments. Lithium treatment partially rescues the cerebellar defects in both zebrafish *arl13b* (Fig. 6) and mouse *Ahi1* mutants [2], suggesting that Wnt signaling is a conserved key pathway regulating early cerebellar development and could serve as a common potential therapy target in JS. Atoh1 and Wnt signaling might only partially contribute to the mechanisms of Arl13b signaling since Arl13b has been found to be distributed subcellularly in the cilium and cytoplasm and is expressed in many tissues and cells.

Most JS research has focused on granule cells and has seldom dealt with Purkinje cells. In human JS samples, the migration of cerebellar Purkinje cells is faulty, with heterotopic and locally-interrupted Purkinje cell layers [42]. The malformation of the Purkinje cell monolayer has also been found in JS gene mutant mice [3, 43]. These late Purkinje cell defects could be due to an early developmental deficiency. It has been demonstrated that mutations of the JS gene *Zfp423/ZNF423* in the mouse impair the early development of Purkinje cell progenitors [44]. Our results also demonstrated that the progenitors of Purkinje cells were selectively disrupted in the dorsomedial cluster in *arl13b*-defecient embryos (Fig. 4), like the early defects in granule cell progenitors. The coincident selective defects of Purkinje and granule cell progenitors suggest that these two populations interact or share common mechanisms. Radial glia and the Bergmann glial cells derived from them provide a scaffold for the migration of both Purkinje cells and granule cells early in cerebellar development [45, 46]. Ciliary Arl13b has been shown to be critical for the polarization of the radial glial scaffold and the laminar organization of neurons in mouse cerebral cortex [6]. The available evidence and our current results suggest that the role of Arl13b in neural development is conserved between species and Arl13b participates in the polarization of the radial glial scaffold as well as coordinating the proliferation and migration of both Purkinje cells and granule cells early in cerebellar development. The migration of Purkinje cells and granule cells also shares molecular mechanisms, such as Reelin signaling [45], the expression level of which was also selectively reduced in the dorsomedial cerebellum in *arl13b*-deficient embryos (Fig. 3).

## Conclusions

We have established a JS model using *arl13b*-deficient zebrafish, which recapitulate some of the signs of JS, such as locomotor and cerebellar developmental defects. The disruption of *arl13b* resulted in a dramatic reduction of granule cells specifically in the CCe, a structure homologous to the human cerebellar vermis. The expression of the proneural gene *atoh1* in a subpopulation of granule cells was down-regulated in the cerebellum. The early development of Purkinje cells was also selectively disrupted in the dorsomedial cerebellum. *wnt1*, a gene transiently expressed early in cerebellar development, was dramatically down-regulated. Furthermore, activating Wnt signaling mitigated the granule cell defects caused by *arl13b*-disruption. Our results reveal the critical role of *arl13b* in the early development of cerebellar granule and Purkinje cells. We propose that the *arl13b*-deficient zebrafish can serve as a powerful tool to investigate the pathological mechanisms underlying JS.

## Electronic supplementary material

Below is the link to the electronic supplementary material.

Supplementary material 1 (PDF 607 kb)

Supplementary material 2 (MP4 25275 kb)
