# Supplementary material for: The Joubert Syndrome Gene arl13b is Critical for Early Cerebellar Development in Zebrafish
Source: Neurosci Bull. 2020 Aug 18;36(9):1023–34. doi: 10.1007/s12264-020-00554-y (PMC7475164; doi:10.1007/s12264-020-00554-y)
Supplement: Supplementary file 1 — Supplementary material 1 (PDF 607 kb) [file 12264_2020_554_MOESM1_ESM.pdf]

# Supplementary Materials

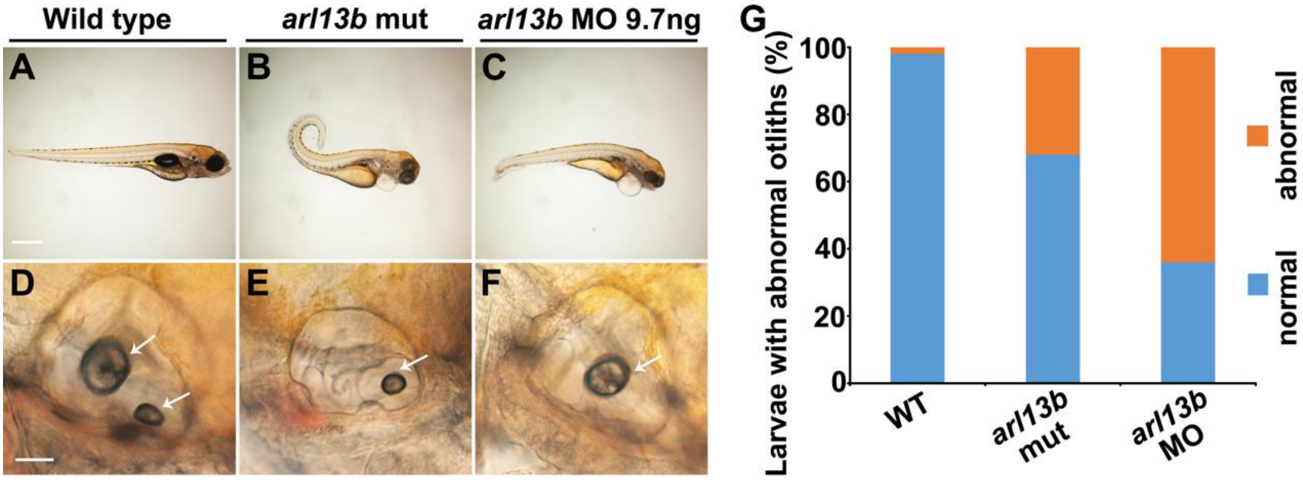


**Fig. S1** Disruption of *arl13b* results in morphological defects. **A**–**C** Wild-type embryos are straight at 4 dpf while the bodies of *arl13b* mutants and morphants are curved. **D**–**G** There are usually two otoliths in the inner ear; however, the otoliths of *arl13b*-deficient embryos have defects in number, size, and morphology. A-C, Scale bar 400 µm. F-I'', D-F, Scale bar 100 µm.

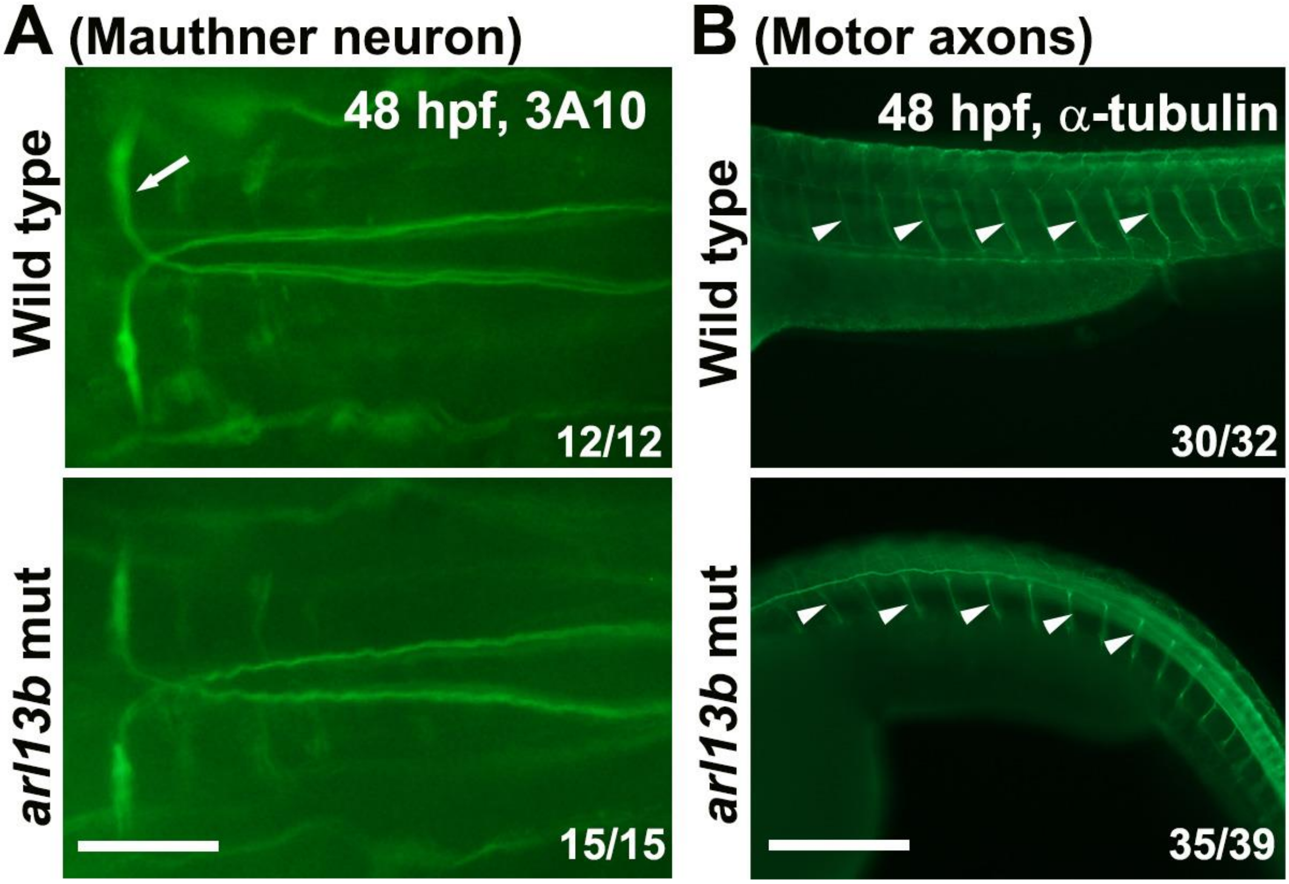


**Fig. S2** Locomotion-related neurons remain intact in *arl13b*-deficient embryos. **A** Mauthner neurons still grow out axons, which normally cross the midline and project caudally, in *arl13b*-mutants. **B** No defect of motor axons is detectable in *arl13b*-mutants. A-B, Scale bar 100 μm.

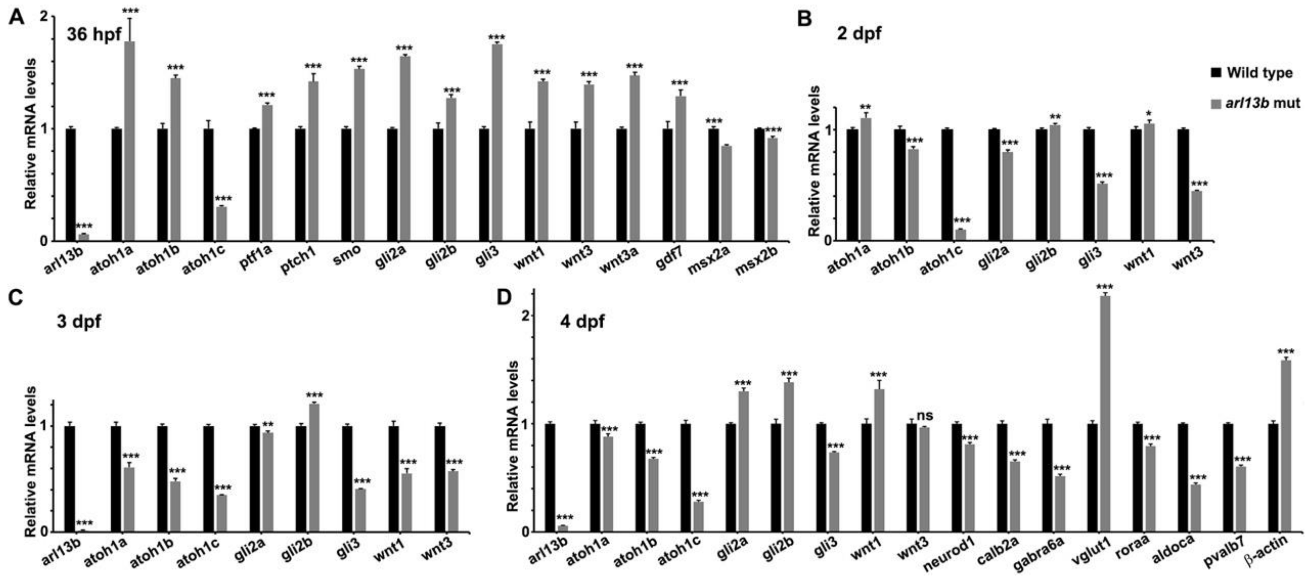


**Fig. S3** The expression levels of a network of genes are altered dynamically in *arl13b* mutants. Total RNAs were extracted from wild type and *arl13b* mutant embryos (whole mount) and reversely transcribed. The obtained cDNAs were used as templates for real time quantitative PCR (q-PCR). The expression levels of the genes are normalized to that of elongation factor EF1α. *arl13b* is significantly reduced in mutants, confirming that the embryos picked on the basis of curved tails are virtually *arl13b* mutants with high penetration, consistent with published data. The expression levels of *atoh1a* and *1b* (granule cell precursors) and *ptf1a* (Purkinje cell precursors) are dynamically regulated in *arl13b* mutants. The expression level of *atoh1c* continually declines in *arl13b* mutants at all developmental stages. The expression levels of Shh signaling (*pcht1*, *smo*, *gli2a*, *gli2b*, and *gli3*), Wnt signaling (*wnt1*, *wnt3*, and *wnt3a*), and BMP signaling (*gdf7*, *msx2a*, and *msx2b*) are altered by *arl13b* deficiency. The molecules expressed in differentiated granule cells (*neurod1*, *calb2a*, *gabra6a,* and *vglut1*) and Purkinje cells (*roraa*, *aldoca*, and *pvalb7*) are also altered. $^{*}P < 0.05$, $^{**}P < 0.01$, $^{***}P < 0.001$.

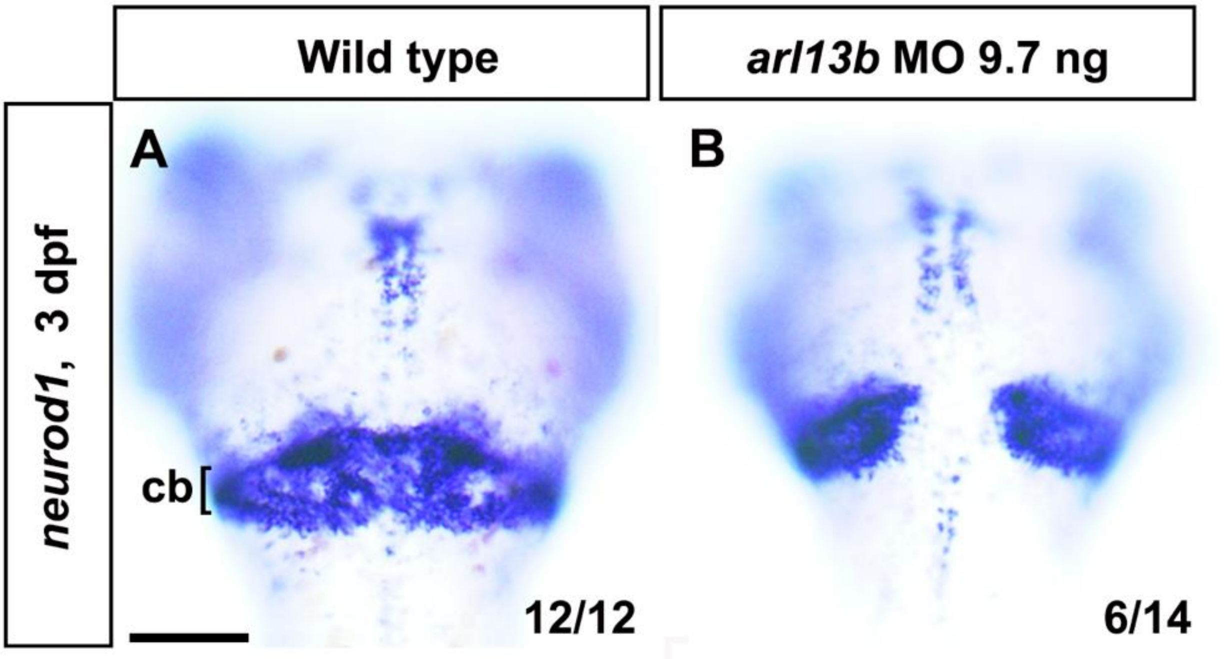


**Fig. S4** The expression of granule cell marker *neruod1* is specifically reduced in the dorsomedial clusters. The dorsomedial clusters of cerebellar granule neurons are specifically impaired by *arl13b* disruption. A-B, Scale bar 100 μm.

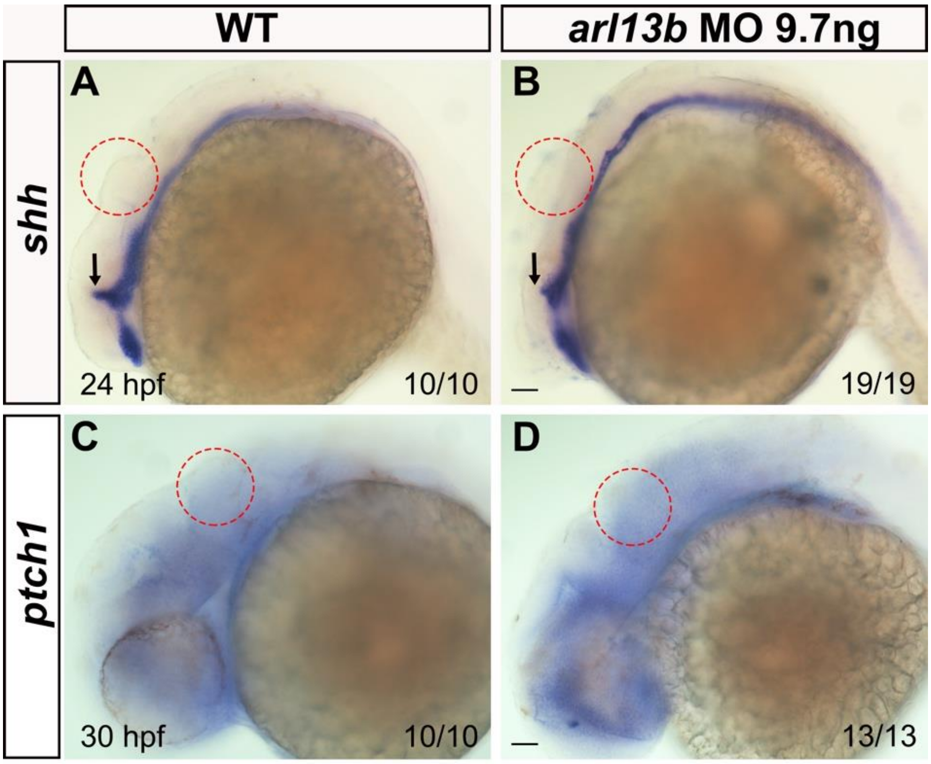

**Fig. S5** The expression of *shh* and its receptor, *patched1* (*ptch1*), is not altered by *arl13b* knockdown. **A, C** The expression of Shh signaling pathway components, *shh* and *ptch1*, is barely detectable in the cerebellum (dashed circles) by *in situ* hybridization in wild-type embryos. **B, D** Their expression is not altered by *arl13b* morpholino treatment although the expression of *shh* is changed in the dorsal zona limitans intrathalamica (arrows). A-D, Scale bar 100 μm.

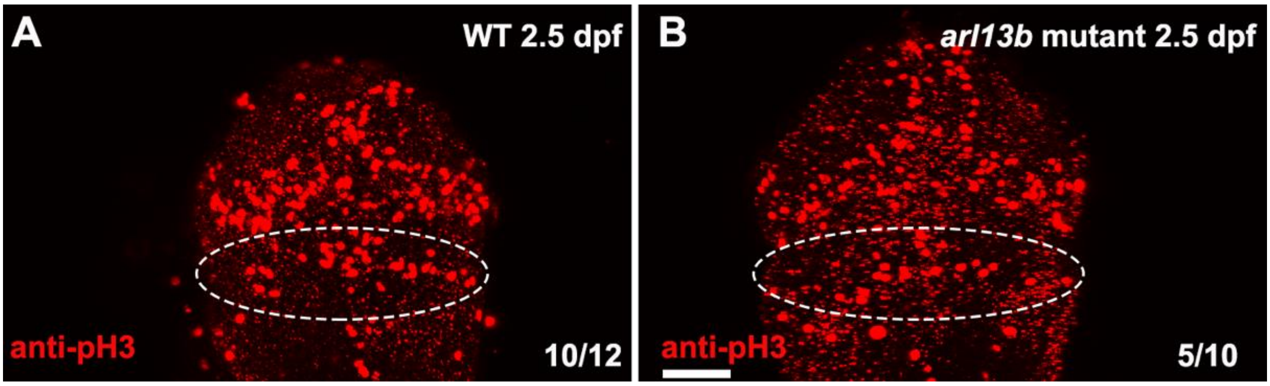


**Fig. S6** The proliferation of cells in the cerebellum is impaired in *arl13b* mutants. **A**, **B** Representative images of wild-type and *arl13b* mutant embryos immunostained with anti-pH3 antibody. The cerebellum is outlined by the dashed oval. A-B, Scale bar 100 μm.

## Supplemental Tables

**Table S1** PCR primers for amplifying gene-specific products

| Gene | Primers | Size (bp) |
|---|---|---|
| *atoh1b* | AAGCGTCATTCCTTCATTGGAG<br>ACAAGTATTGACGTGTGCTGAG* | 402 |

| | | |
|---|---|---|
| *atoh1c* | ATGCCCCATCCGGACACCCCTTTTGG<br>CTATTTTACACCATTGTTCCTTTCCA* | 615 |
| *neurod1* | TTTGGGCTCTTTCGGAAATCTTG<br>AGACCCGCTGCCTGATAGTG* | 469 |
| *wnt1* | CCCAAACTAACAAGTCAGC<br>AAGCCCTCCCTATTTACCAC* | 947 |
| *ptch1* | ATACCAAGTGTTGCATGGTG<br>AGCTCATTAAACGGCAAGAG* | 638 |
| *zic1* | GAAAATCTGAAAATCCACAAAA<br>GGTGCTGTGGCTGCTCGTGT* | 365 |

* T3 promoter sequence (GGATCCATTAACCCTCACTAAAGGGAA) is added to the 5'-end of the reverse primer for transcription of the gene *in vitro*.

**Table S2** PCR primers for quantitative real-time PCR.

| Gene | Primer |
|---|---|
| *β-actin* | CGGTTTTGCTGGAGATGATGC |
| | TGGGGTATTTGAGGGTCAGGA |
| *arl13b* | GGAGAGAACCTGCAAGGAAGG |
| | GATTCCTCTGACTGTCGCCG |
| *atoh1a* | CCCAGGCAAAATATCCGTCCCT |

| | |
|---|---|
| | CAAGGCTGGTGGGTACTCGCT |
| *atoh1b* | CCTCAAGCTGCCTCTGAAGA |
| | GGGCCATCTGAAGAGTGTCA |
| *atoh1c* | CCCGAGAGAGGAGGAGGATG |
| | GGGTATGGGGTTTCTGGGTC |
| *neurod1* | CGAGCAGAGCCAGGAGAT |
| | AGGGTGGTGTCAAAGAACG |
| *vglut1* | CTGTGGAGGGTTTGGAATGGA |
| | GCGATGTCAAGATGGTTCACA |
| *gabra6b* | TCTTGTGTTGGACAGGTGGTG |
| | AGGTCGGAGTCTGTTGTCATATC |
| *ptf1a* | TGCGATCTCACATCCCAACA |
| | CCGTAGTCTGGGTCATTTGGA |
| *roraa* | TGGCCCAGAACATTTCCAAGT |
| | TGGCAAACTCCACCACATACT |
| *pvalb7* | ATCCTGAGTGAGACCGAGCT |
| | TGTGGTCGAAACTGTCAGCA |
| *calb2a* | CCTTACCTTCACTTGGCGGA |
| | GCTCCTCTCCTCGCAATCTC |
| *aldoca* | GCGTCCCGTTTGTCAAGATG |
| | GGCAAAGTCAGCTCCATCCT |
| *gdf7* | AACACGGTCACCAGCTTCAT |

| | |
|---|---|
| | GCAGCTCCACTTCCATCAGT |
| *msx2a* | AGGAGCGAGTCAACTTCACG |
| | CAAGAGCTGAGCAGTGGTGA |
| *msx2b* | AGAAGGCGAGAGGATGTGTCCAG |
| | GATTGGTCTTGTGCTTGCGGAG |
| *wnt1* | ATGTGTCCTCCTGGTGTCCTCTC |
| | CTGGCGGCGACTTAGCAGTG |
| *wnt3* | AGCAGCAGCAAACCTTGGAG |
| | CCCGAGAGCTGGAGAACCAC |
| *wnt3a* | CTCCGCTTCTGCCGCAACTAC |
| | CTGATGCTGACACTCCTGGATGC |
| *gli2a* | CAGCACCAGCAGCAGCACTG |
| | CCTTGGACCGCTTGAAGATCATGG |
| *gli2b* | GCGGTCAGCAGCACAGTCAAC |
| | ACAGTCGTCCATGTCCTCCTTCAG |
| *gli3* | TTGCCATGGACCCTCGTTCT |
| | GGTACTGGCGAGGGTTCGTA |
| *ptch1* | TGCGCTGGAGCAGATATCAGAGG |
| | TCCACGTCCGTCTCCAGATTAGC |
| *smo* | TGGCATTTGCTTTGTCGGCT |
| | CAGCCCTGGATGGTTGCTTTTA |
